# COVID-19 quarantine in chronic kidney disease patients: A focus on sarcopenia traits

**DOI:** 10.1590/2175-8239-JBN-2020-0201

**Published:** 2021-03-22

**Authors:** Heitor Siqueira Ribeiro, Kenneth R. Wilund, Ricardo Moreno Lima

**Affiliations:** 1 Universidade de Brasília, Faculdade de Educação Física, Brasília, DF, Brasil.; 2 University of Illinois at Urbana-Champaign, Department of Kinesiology and Community Health, Urbana, IL, USA.

**Dear Editor,**

Recently, the Brazilian Journal of Nephrology (BJN) published a supplementary issue regarding the Coronavirus Disease 2019 (COVID-19) impact on clinical nephrology routine, with a special focus on people with chronic kidney disease (CKD)^1^. We congratulate the BJN for the initiative, as it will have a high impact on COVID-19 management for nephrology professionals. Nonetheless, we would like to call attention to a negligence about the COVID-19 quarantine impact on physical function and musculoskeletal health, which has not been introduced and discussed by the BJN.

People with CKD usually have high levels of sedentary behavior, which increases across the stages of the disease. Wilkinson et al. observed that walking is the most popular form of physical activity of people with CKD^2^, so greater sedentary behavior is expected as a consequence of COVID-19 quarantine. Hence, losses in muscle mass and strength are also expected, which is known to negatively affect physical function in this population^3^. Moreover, Cheval et al. found that an increase in sedentary behavior during COVID-19 quarantine was associated with poorer physical health, mental health, and subjective vitality in general subjects^4^.

**Figure 1:**
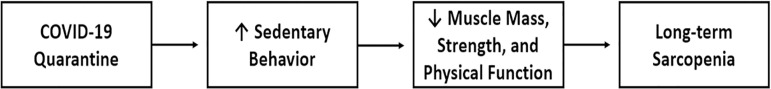
COVID-19 quarantine and its association with sarcopenia in chronic kidney disease patients.

Sarcopenia is defined by an age-related decline in muscle mass, strength, and physical function. It is known that people with CKD are at higher risk for sarcopenia, which is related to a systemic catabolic state, higher protein energy wasting, and other metabolic disorders^5^. As seen in Figure 1, quarantine-related muscular disuse and inadequate dietary intake may potentially increase sarcopenia signs among people with CKD, a population that already presents with reduced functional reserve.

People with CKD not yet in hemodialysis (HD) may be experiencing an even greater impact from quarantine than HD patients. In general, HD patients are still attending their dialysis clinics for scheduled treatments, so social isolation may not have impacted their routines to the same extent. Conversely, non-dialysis CKD patients do not have the same constraints, and they are generally more physically active. Thus, quarantine and social isolation due to COVID-19 may have a greater impact on their current lifestyle. The duration of social distancing that will be required for high risk individuals is not known, but the longer it lasts, the greater the impact it will likely have on the development and progression of sarcopenia in people with CKD around the world. Related consequences could include increases in cardiovascular events, hospitalization, progression to renal failure, mortality, and poor prognosis for kidney transplants. Thus, the European Work Group on Sarcopenia in Older People (EWGSOP2)^6^ criteria should be used for screening and monitoring sarcopenia (Table 1), as well as SARC-F questionnaire in the absence of direct measures.

**Table 1:** Sarcopenia screening and cut-off points according to the european work group on sarcopenia in older people (Ewgsop2)

Tests	Cut-off points for men		Cut-off points for women
**EWGSOP2 sarcopenia cut-off points for low strength**			
Handgrip Strength	<27 kgf		<16 kgf
Sit-to-stand	>15s for five repetitions		>15s for five repetitions
If low strength is confirmed, a probable sarcopenia is confirmed with muscle quantity			
**EWGSOP2 sarcopenia cut-off points for low muscle quantity**			
ASMM	<20 kg		<15 kg
ASMM/height^2^	<7.0 kg/m^2^		<6.0 kg/m^2^
If low muscle quantity is verified, sarcopenia is confirmed and severity is analyzed			
**EWGSOP2 sarcopenia cut-off points for low performance**			
Gait Speed		≤0.8 m/s	
Short Physical Performance Battery		≤8 point score	
Timed-Up and Go		≥20 s	
400m walk test		Non-completion or ≥6 min for completion	
If low performance is seen, severe sarcopenia is confirmed			

ASMM = Appendicular Skeletal Muscle Mass; EWGSOP2 = Revised European Work Group on Sarcopenia in Older People.

Health professionals involved in the management of CKD need to consider strategies in order to mitigate the adverse effects of quarantine and social distancing on physical activity and musculoskeletal health. Exercise and nutrition interventions can potentially attenuate these adverse effects. Therefore, we encourage patients of all CKD stages to maintain or engage an active lifestyle, as well as adequate dietary intake during COVID-19 quarantine. To make these interventions safe and feasible, we recommend two guides: Coronavirus Disease 2019: Quick Diet and Nutrition Guide for Patients With Chronic Kidney Disease (https://www.jrnjournal.org/article/S1051-2276(20)30213-2/fulltext) and My Get Active Guide (http://move.bangor.ac.uk/get-active.php.en), both available in Portuguese and English versions.
